# Supplementary material for: Content-rich biological network constructed by mining PubMed abstracts
Source: BMC Bioinformatics. 2004 Oct 8;5:147. doi: 10.1186/1471-2105-5-147 (PMC528731; doi:10.1186/1471-2105-5-147)
Supplement: Additional File 2 — The original results of the above study (non-essential files are deleted to keep the file size under the limit set by BMC bioinformatics). [file 1471-2105-5-147-S2.bz2 › chilibotAdditionalFile2/dip10/93ID8950973E400/html/GRB2_KIT.html]

 


 **GRB2** and **KIT** 
  
Found 20 abstracts in PubMed, retrieved 10.  
 

 What does Google say? 
 PDF only 
| .edu only 

---

**Interactive relationship** (e.g. stimulation, inhibition, etc)

**Stimulatory relationship**- These data indicate that the synergistic cross talk between SCF and ET 1 signaling is initiated through the pathway of tyrosine phosphorylation of c  **kit**   [ **KIT** ] , which results in the enhanced formation of the Shc Grb 2  [ **GRB2** ]  complex which leads in turn to the synergistic activation of the Ras Raf 1 MEK MAP kinase loop.  Ref: 10921922 J Biol Chem, 2000
**Neutral relationship**- The binding specificity of the Gads SH2 domain is similar to  **Grb2**  and mediates the interaction of Gads with Shc, Bcr Abl and c  **kit**   [ **KIT** ] .  Ref: 9872323 Oncogene, 1998

**Non-interactive relationship** (e.g. studied together, co-existance, homology, etc.)

- In order to define the mechanism by which Socs1 mediated suppression of  **Kit**  dependent mitogenesis occurs, we demonstrate that Socs1 binds to the signalling proteins Grb 2  [ **GRB2** ]  and the Rho family guanine nucleotide exchange factors Vav.  Ref: 10022833 EMBO J, 1999
- Stimulation of  **Kit**  by its ligand, stem cell factor activates several distinct early signaling pathways, including PLC gamma, PI 3Kinase, Src kinase,  **Grb2**  and Grb7.  Ref: 12486028 J Biol Chem< MedlineTA>J Biol Chem, 1999
- A GST fusion protein containing the SH3 domain of the  **Grb2**  adaptor protein does not inhibit tr  **kit**  induced egg activation, showing that the effect of the SH3 domain of PLCgamma1 is specific.  Ref: 9722617 J Cell Biol, 1998
- Binding of  **Grb2**  to Tyr 703 in the c  **Kit**   [ **KIT** ]  SCFR  [ **KIT** ]  provides a link to the Ras mitogen activated protein kinase pathway.  Ref: 10377264 Biochem J, 1999
- Identification of Tyr 703 and Tyr 936 as the primary association sites for  **Grb2**  and Grb7 in the c  **Kit**   [ **KIT** ]  stem cell factor receptor  [ **KIT** ] .  Ref: 10377264 Biochem J, 1999
- Combined stimulation with Epo also resulted in impaired cooperation between Src defective  **Kit**  mutant and Epo R, and to a lesser extent with  **Kit**  mutants defective in the activation of PI 3Kinase or  **Grb2** .  Ref: 12486028 J Biol Chem< MedlineTA>J Biol Chem, 1999
